# The effect of stress perceived in clinical practice on professional behavior in nursing students: a correlational and descriptive study

**DOI:** 10.1007/s11845-025-03989-2

**Published:** 2025-06-27

**Authors:** Nurten Gülsüm Bayrak, Elif Güzide Emirza

**Affiliations:** 1 Faculty of Health Sciences, Department of Nursing/Psychiatric Nursing, Giresun University, Giresun, Türkiye; 2 Faculty of Health Sciences, Department of Nursing/Psychiatric Nursing, Ondokuz Mayıs University, Samsun, Türkiye

**Keywords:** Clinical practice, Descriptive study, Nursing student, Perceived stress, Professional behavior

## Abstract

**Background:**

Perceived stress in clinical practice in nursing students affects their professional behaviors.

**Aims:**

Perceived stress in clinical practice in nursing students affects their professional behaviors. The study examined the effect of stress perceived by nursing students in clinical practice on professional behavior.

**Methods:**

The correlational and descriptive study population consisted of nursing students studying at a state university in northern Türkiye, and the sample consisted of 2nd, 3rd, and 4th year nursing students. “Personal Information Form,” *“*Nursing Students Professional Behaviors Scale,” and “Perceived Stress Scale for Nursing Students” forms and scales were used to collect the data. Descriptive statistics, Pearson correlation test, Mann–Whitney U test, Kruskall Wallis test, and linear regression analysis were used to analyze the data.

**Results:**

A significant relationship was found between perceived stress and professional behavior in nursing students who participated in the study. In addition, there was a significant relationship between the mean scores of gender, class, reason for choosing nursing, liking the nursing profession and clinical practice, and smoking variables and the mean scores of perceived stress and professional behavior. It was also concluded that perceived stress had a predictive effect on professional behavior and sub-dimensions (*p* < 0.005).

**Conclusions:**

This study revealed that perceived stress in nursing students is effective in professional care behavior. Thus, it provides essential findings for increasing stress management and professional behaviors in nursing education. In this context, improvements in educational processes and strengthening support mechanisms to reduce the negative effects of stress will contribute to the professional development of nursing students.

## Introduction

Nursing is a professional profession that can be revised with socio-cultural and technological innovations. It provides professional health services to individuals, families, and society and is essential in improving health [25]. Nursing education requires professional knowledge and skills and consists of theory and practice [35]. In this context, clinical practice is of great importance in transforming theoretical knowledge into practical skills and in the professional development of students [16]. In clinical practice, students’clinical practice and skills are monitored by universities, and activities related to the integration of academic knowledge into practice are evaluated [19]. In the clinical practice process of nursing education, students develop their clinical skills, have the opportunity to apply theoretical knowledge, develop problem-solving skills, and make clinical reasoning [39]. In addition, studies report that clinical practice experiences facilitate the transition to working life, especially in senior students, and strengthen commitment to the profession [15, 38].

Clinical practices also pave the way for problems, such as the complex structure of the clinical environment, intense work tempo, and different multidisciplinary approaches in patient care activities, contrary to many positive gains. In addition, unprofessional attitudes, inconsistencies regarding clinical practices, interpersonal communication, and crowded student groups are other problems [32, 36, 46]. In studies conducted in this context, it has been reported that students have problems interacting with patients. Factors such as fear of making mistakes, lack of experience, and supervision may cause students to perceive the clinical practice environment as unsafe, which may cause anxiety and stress in students [13, 40].

The components of the professional nursing practice have been identified as providing care in line with patient interests, humanism, social responsibility, sensitivity to culture and beliefs, high competence and knowledge, and attention to ethical standards [7]. Many factors, such as students’anxiety about academic failure, theoretical and practical differences, ethical dilemmas, burnout, poor relationships between instructors, etc., can increase anxiety and stress, negatively affect self-confidence and view of the profession, and interrupt students’professional approach in clinical practice [2, 17, 28, 34, 41].

Nursing education occurs in clinical practice, where students acquire professional skills, which is a significant source of stress. This process directly affects both the academic and professional development of students. Although there are many studies in the literature on the psychological, physical, and academic effects of stress experienced by nursing students in clinical practice [31, 32, 48, 49] research on the specific effects of this stress on professional behaviors is limited. For example, Stutzer et al. [48] state that clinical stress hurts students’general psychological health, while Yılmaz [49] emphasizes the negative effects of stress on academic achievement and learning [40, 48, 49]. However, these studies do not examine in depth the effects of stress on professional behaviors, such as ethical decisions, empathy, communication skills, reporting, practice skills, and professionalism. The development of nursing students’professional behaviors is directly related to the stress they encounter in clinical experiences, and understanding this relationship is critical for improving nursing education [44]. In this context, this gap in the existing literature reveals the importance of this study. The study aims to understand the effects of stress perceived by nursing students in clinical practice on their professional behaviors. It also aims to develop strategies for improving the educational processes by investigating the relationship between students’ stress-coping mechanisms and professional behaviors.

This study aims to fill an important gap in the literature to understand nursing students’difficulties in forming their professional identities and to develop more effective educational methods in this process. Thus, it will contribute to the current understanding of nursing education and identify the support needed for students to exhibit professional behavior more effectively in the clinical setting. The study’s results can be considered a stepping stone to reveal the problems. However, the results obtained are also significant regarding efficient and effective clinical practice and revealing the obstacles in forming the perspective and commitment to the profession.

## Material and method

### Study design

**Fig. 1:**
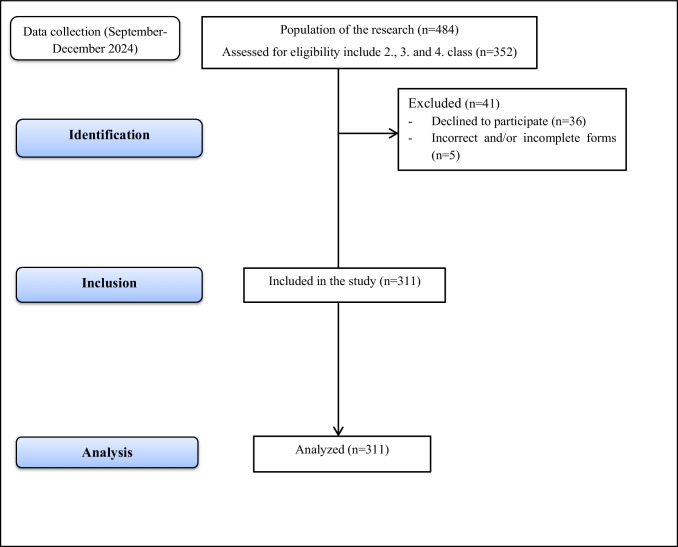
STROBE flow chart. STROBE, (strengthening the reporting of observational studies in epidemiology)

This study was a correlational descriptive study. Strengthening the Reporting of Observational Studies in Epidemiology (STROBE) was used to report the study (Fig. 1).

#### Participants, variables, and study size

The research data were collected between September and December 2024 from 2nd, 3rd, and 4th year nursing students at the Department of Nursing of a university in the province of Türkiye. Since the effect of perceived stress in clinical practice on the professional behavior of nursing students was investigated in the study, first-year students who had not yet started practice were not included in the sample. In the university where the data were collected, there are 484 nursing students, including 1 st, 2nd, 3rd and 4th grades. The sample size was calculated according to the known sample size calculation. According to this calculation, the sample size was 215 nursing students with a 5% margin of error and 95% confidence interval. However, considering the risks, such as incomplete and incorrect completion of forms and scales, this number was increased by 20%, and the sample size was determined as 258 nursing students [9]. More nursing students were reached than the determined sample size, and the study was completed with 311 participants. In the post hoc analysis, considering the mean scores of the Nursing Students Professional Behaviors Scale (NSPBS) and Perceived Stress Scale for Nursing Students (PSSNS), the effect size of the sample was found as d = 2.124 using G*Power 3.1.9.7 program. According to Cohen, effect size (d) values are considered weak if d = 0.2, medium if d = 0.5, and high if d = 0.8. The fact that the sample in the study has an effect size of d = 2.124 shows that the sample size reached is very high. The inclusion and exclusion criteria for participation in the study are as follows:

*Inclusion Criteria:*

- To be a nursing student,
- To be studying in one of the 2nd, 3rd, and 4th grades of the nursing department,
- 18 years of age or older,
- Volunteering for the research,
- Complete data collection forms in a comprehensive manner.

Exclusion Criteria:

- Mental and/or factual impairment,
- Filling in forms and scales incompletely,
- It is the willingness to withdraw from work.

#### Data sources/measurement

The data were obtained by using "The Personal Information Form," "Nursing Students Professional Behaviors Scale (NSPBS)," and "Perceived Stress Scale for Nursing Students (PSSNS). "Filling out the forms and scales took approximately 15–20 min.

##### Personal information form

The researchers created it to determine the socio-demographic characteristics of nursing students in line with the relevant literature [8, 18, 31]. The form includes eight questions to determine age, gender, high school graduate, attitude towards the nursing profession, smoking, and alcohol use.

##### Nursing students professional behaviors scale (NSPBS)

It was developed by Goz and Geçkil in 2010 to determine the professional behaviors of nursing students. Content and construct validity were examined for the scale’s validity; internal consistency and item-total score correlations were examined for reliability. In the factor analysis of the scale, factor loadings ranged between 0.38–0.79, and 3 sub-dimensions with eigenvalues exceeding one were obtained. The sub-dimensions are as follows:

1. Health Care Practices Dimension (items 1,3,4,6,8,8,9,10,10,12,16,17,18,19,20,21,23,25,26,27)
2. Activity Applications (items 2,5,7,11,13,14,15)
3. Reporting dimension (Articles 22 and 24)

The three factors collectively explain 56.46% of the variance. According to the scale’s total score, Cronbach’s alpha reliability coefficient is 0.95, and item-total score correlations are between 0.42 and 0.80 (*p* < 0.01). The scoring of the items on the scale is "Quite Adequate" "= 5,"Partially Adequate" "= 4, "Undecided" "= 3,"Inadequate" "= 2, and"Extremely Inadequate" "= 1. The scores that can be obtained from the scale vary between 27 and 135; the higher the scale score, the higher the students’ability to apply professional behaviors (Goz, Geçkil, 2010). In our study, Cronbach alpha values of the scale were found to be 0.92 for the total scale score, 0.90 for the healthcare practices dimension,

0.82 for the Activity practices dimension and 0.79 for the Reporting dimension.

##### Perceived stress scale for nursing students (PSSNS)

Initially developed by Sheu et al. [40] in Chinese, the scale consists of 29 items. The Turkish validity and reliability study was conducted by Karaca et al. [21]. In evaluating the items, a five-point Likert-type scale was used as’’4- Very stressful for me, 3, 2, 1, 0- Not stressful for me’’. Sub-dimensions of the scale:

1. Stress due to lack of professional knowledge and skills; 2, 7, 11
2. Stress experienced while caring for the patient; 1, 4, 6, 8, 10, 12, 14, 19
3. Stress caused by homework and workload; 3, 9, 13, 17, 21
4. Stress caused by instructors and nurses; 5, 16, 18, 20, 25, 27
5. Environmental stress; 5, 23, 26
6. Stress from peers and daily life; 22, 24, 28, 29

The total score varies between 0–116. A high score indicates high stress [21]. In our study, the cronbach alpha value of the scale over the total score was found to be 0.97.

### Ethical consideration

This study was approved by Giresun University Rectorate Social Sciences, Science and Engineering Sciences Research Ethics Committee and the institution where the application was conducted with the protocol number E-50288587–050.01.04–15625 dated 05.06.2024. In addition, institutional permission was obtained from the university’s Dean of the Faculty of Health Sciences. All volunteers participating in the study were informed about the purpose and nature of the study, and consent was obtained for participation. The principles of the Declaration of Helsinki conducted each stage of the study. In addition, to prevent bias in the research, the participants were explained about the research and the confidentiality of the data, and the participants themselves filled in the forms.

### Statistical methods and quantitative variables

Data were analyzed using SPSS (Statistical Package for the Social Sciences, Inc., Chicago, IL, USA) version 25.0. Numbers, percentages, means, and standard deviations were used for descriptive statistics. The normal distribution of the variables was evaluated with the Kolmogorov–Smirnov test, and it was determined that the variables and scale mean scores were normally distributed. The Pearson correlation test was used to evaluate the difference between the scales. Since it was seen that the variables were not normally distributed, nonparametric tests were used. In this context, the Mann–Whitney U test was used to compare the averages and scale scores between the two groups. Kruskall Wallis test was used to compare the averages and scale scores between three or more groups. Linear regression analysis was used to determine the predictive factors of perceived stress on professional behavior. A significance level of *p* < 0.05 was accepted for all analyses.

## Results

### Descriptive characteristics of nursing students

The mean age of the participants (n = 311) was 21.53 ± 2.29, and 76.4% (n = 253) were female. Of the participants, 38.4% (n = 127) were in 4th grade, and 65% (*n* = 215) were graduates of Anatolian High School. Of the nursing students, 52.6% (*n* = 174) stated that they chose the nursing profession because of their desire, and 63.1% (*n* = 209) stated that they enjoyed the nursing profession and clinical practice. It was found that 78.9% (*n* = 261) of the participants did not smoke, and 82.8% did not drink alcohol (Table 1).

**Table 1:** Demographic characteristics of nursing students participating in the study (*n* = 331)

	*n*	%	Age (**x̄ ± SD**)	**Min**	**Max**
– Female	253	76.4	21.53 ± 2.29	17	38
– Male	78	23.6			
**Classroom**	*n*	**%**	**High school graduated**	*n*	**%**
– Class 2	114	34.4	– Health Vocational High School	25	7.6
– Class	90	27.2	– Anatolian High School	215	65.0
– Class	127	38.4	– Science High School	51	15.4
			– Other	40	12.1
**Reason for choosing nursing**	*n*	**%**	**Liking the nursing profession and clinical practice**	*n*	**%**
– By his own request	174	52.6	– Yes	209	63.1
– By family request	60	18.1	– No	36	10.9
– Teacher guidance	11	3.3	– Undecided	86	26.0
– Other	86	26.0			
**Cigarette use**	*n*	**%**	**Alcohol use**	*n*	**%**
– Yes	70	21.1	– No	274	82.8
– No	261	78.9	– Sometimes use	53	16.0
			– Regular use	4	1.2

### The relationship between NSPBS and PSSNS scores and scales of nursing students participating in the study

When the arithmetic mean and standard deviation values for the total and sub-dimension scores of the NSPBS are examined,"Health Care Practices"79.71 ± 5.46,"Activity Applications,"30.07 ± 3.22,"Reporting,"8.78 ± 1.24,"Total"123.36 ± 9.05. When the arithmetic mean and standard deviation values related to PSSNS scores are examined,"-Stress due to lack of professional knowledge and skills"7.03 ± 43.31,"Stress while caring for the patient"19.24 ± 8.35,"Stress caused by homework and workload"12.22 ± 5.25,"Stress caused by lecturers and nurses"13.74 ± 6.36,"Stress from the environment"6.98 ± 3.25,"Stress from peers and daily life"9.16 ± 4.36 and"Total"68.53 ± 29.12 (Table 2).

**Table 2:** NSPBS and PSSNS total and subscale scores of nursing students participating in the study (*n* = 331)

Scale and Subscale	Mean (x̄)	Std. Deviation (SD)	Min	Max
Total NSPBS	123.36	9.05	91.00	135.00
– Health Care Practices	79.71	5.46	60.00	85.00
– Activity Applications	30.07	3.22	22.00	35.00
– Reporting	8.78	1.24	6.00	10.00
Total PSSNS	68.53	29.12	0.00	116.00
– Stress due to lack of professional knowledge and skills	7.03	3.31	0.00	12.00
– Stress while caring for the patient	19.24	8.35	0.00	32.00
– Stress caused by homework and workload	12.22	5.25	0.00	20.00
– Stress caused by lecturers and nurses	13.74	6.36	0.00	24.00
– Stress from the environment	6.98	3.25	0.00	12.00
– Stress from peers and daily life	9.16	4.36	0.00	16.00

*NSPBS* Nursing Students Professional Behaviors Scale, *PSSNS* Perceived Stress Scale for Nursing Students

A low and negative correlation (r = −0.165, p = 0.003; r = −0.162, p = 0.003; r = −0.160, p = 0.004; r = −0.165, p = 0.003; r = −0.166, p = 0.003; r = −0.153, *p* = 0.005; r = −0.127, *p* = 0.021) was found between the participant’s total NSPBS scores and total PSSNS and all subscale scores. A low level and negative correlation (r = −0.112, *p* = 0.042; r = −0.122, *p* = 0.027) was found between "the Health Care Practices" sub-dimension scores and "Stress due to lack of professional knowledge and skills" and "Stress caused by lecturers and nurses" scores. In the study, there was a moderate and low level and negative correlation between the  "Activity Applications" sub-dimension scores of the participants and total PSSNS and all sub-dimension scores (r =—0.251, *p* < 0.001; r = −0.241, *p* < 0.001; r = −0.259, *p* < 0.001; r = −0.256, *p *< 0.001; r = −0.235, *p* < 0.001;

r = −0.217, *p* < 0.001; r = −0.217, *p* < 0.001). In addition, there was a low level and negative correlation (r = −0.112, *p* = 0.041) between "Reporting" sub-dimension scores and"Stress caused by homework and workload"scores (Table 3).

**Table 3:** Correlation between the NSPBS and PSSNS

		Total NSPBS		Health Care Practices		Activity Applications		Reporting	
		*p* value*	r	*p* value*	r	*p* value*	r	*p* value*	r
**Sub-dimensions**	Total PSSNS	0.003*	**−0.165**	0.050	−0.108	< 0.001*	**−0.251**	0.205	−0.070
	- Stress due to lack of professional knowledge and skills	0.003*	**−0.162**	0.042*	**−0.112**	< 0.001*	**−0.241**	0.331	−0.054
	- Stress while caring for the patient	0.004*	**−0.160**	0.080	−0.096	< 0.001*	**−0.259**	0.211	−0.069
	- Stress caused by homework and workload	0.003*	**−0.165**	0.080	−0.096	< 0.001*	**−0.256**	0.041*	**−0.112**
	- Stress caused by lecturers and nurses	0.003*	**−0.166**	0.027*	**−0.122**	< 0.001*	**−0.235**	0.451	−0.042
	- Stress from the environment	0.005*	**−0.153**	0.064	−0.102	< 0.001*	**−0.217**	0.168	−0.076
	- Stress from peers and daily life	0.021*	**−0.127**	0.097	−0.091	< 0.001*	**−0.194**	0.630	−0.027

*p < 0.05: Statistically significant, r: Pearson correlation, *NSPBS* Nursing Students Professional Behaviors Scale, *PSSNS* Perceived Stress Scale for Nursing Students

This shows a low and moderate relationship between perceived stress levels and the professional behaviors of nursing students. The perceived stress levels of the participants in the study in the nursing profession and clinical practices create a negative relationship with their professional behaviors. This means that the increase in stress experienced by students in professional and clinical practices may negatively affect professional behavior towards the profession and patients. According to this result in our study, while a strong relationship was expected between the perceived stress levels of nursing students and their professional behaviors, the fact that there is a low and medium-level relationship is a different and more important finding than expected.

#### Comparison of NSPBS and PSSNS scores of nursing students and predictive results

The comparison of NSPBS and PSSNS mean scores and descriptive variables is presented in Table 4. It was found that the mean scores of the students whose gender was "female" in the total and all sub-dimension mean scores of the PSSNS were significantly higher than those of the students whose gender was "male" (p < 0.05). In addition, it was determined that the mean scores of the students whose Reason for choosing to nurse was "Other" from the mean scores of the exact scale total and other sub-dimensions were significantly higher than the others (*p* < 0.05). It was determined that the mean scores of non-smokers were significantly higher than those of smokers in the total and the last three sub-dimensions of PSSNS (*p* < 0.05).

**Table 4:** Comparison of NSPBS and PSSNS scores of the nursing students included in the study according to some variables (*n* = 311)

Variables					Total NSPBS	Health Care Practices Sub-dimension	Activity Sub-dimension	Reporting Sub- dimension	Total PSSNS	Stress due to lack of professional knowledge and skills Sub-dimension	Stress while caring for the Sub-dimension	Stress caused by homework and workload Sub-dimension		Stress caused by lecturers and nurses Sub-dimension	Stress from the environment Sub-dimension	Stress from peers and daily life Sub-dimension
**Gender**	Female			Mean ± SD	123.22 ± 9.15	78.07 ± 12.13	29.97 ± 3.26	8.78 ± 1.23	**73.01 ± 28.23**	**7.46 ± 3.25**	**20.46 ± 8.03**	**12.94 ± 5.15**		**14.73 ± 6.19**	**7.41 ± 3.17**	**9.90 ± 4.21**
	Male			Mean ± SD	123.79 ± 8.75	82.42 ± 12.58	30.38 ± 3.12	8.76 ± 1.28	**53.97 ± 27.31**	**5.65 ± 3.13**	**15.29 ± 8.19**	**9.89 ± 4.91**		**10.53 ± 5.83**	**5.57 ± 3.13**	**6.78 ± 3.98**
Mann–Whitney U test					U = 9567.5	U = 9720.5	U = 9214.0	U = 9838.5	**U = 6193.0**	**U = 6728.5**	**U = 6518.5**	**U = 6586.5**		**U = 6132.0**	**U = 6682.5**	**U = 5836.0**
Z statistic					−0.406	−0.200	−0.888	−0.041	**−4.973**	**−4.268**	**−4.536**	**−4.450**		**−5.063**	**−4.330**	**−5.472**
^*p*value^					*p* = 0.685	*p* = 0.842	*p* = 0.374	*p* = 0.967	***p* < 0.001**	***p* < 0.001**	***p* < 0.001**	***p* < 0.001**		***p* < 0.001**	***p* < 0.001**	***p* < 0.001**
Effect size (d)						–	–	–	**0.6971**	**0.5782**	**0.6438**	–		**0.7204**		
**Classroom**		Grade 2		Mean ± SD	122.60 ± 9.37	**79.04 ± 5.68**	30.14 ± 3.37	8.66 ± 1.28	68.61 ± 27.32	7.19 ± 3.23	19.23 ± 7.93	12.30 ± 4.92		13.64 ± 6.12	7.09 ± 3.13	9.13 ± 4.12
		Grade 3		Mean ± SD	122.60 ± 9.47	**79.28 ± 5.83**	29.76 ± 2.97	8.76 ± 1.27	70.62 ± 27.81	6.88 ± 3.05	19.73 ± 8.11	12.52 ± 5.23		14.36 ± 5.99	7.20 ± 3.08	9.66 ± 4.09
		Grade 4		Mean ± SD	124.58 ± 8.37	**80.62 ± 4.88**	30.22 ± 3.280	8.89 ± 1.20	66.97 ± 31.62	7.00 ± 3.56	18.90 ± 8.91	11.95 ± 5.57		13.40 ± 6.82	6.73 ± 3.49	8.85 ± 4.73
Kruskal Wallis test					3.420	**6.046**	1.162	2.007	0.543	0.488	0.335	0.546		1.342	1.081	1.628
*p* value					*p* = 0.181	***p* = 0.049**	*p* = 0.559	*p* = 0.367	*p* = 0.762	*p* = 0.784	*p* = 0.846	*p* = 0.761		*p* = 0.511	*p* = 0.583	0.443
Effect size (d)					–	**0.9822**	–	–	–	–	**–**	–		–		
**Reason for choosing nursing**		By his own request		Mean ± SD	**124.75 ± 8.99**	**80.40 ± 5.43**	**30.67 ± 3.09**	8.85 ± 1.23	**65.91 ± 29.50**	6.83 ± 3.30	**18.64 ± 8.38**	**11.58 ± 5.43**		**13.26 ± 6.46**	**6.67 ± 3.28**	**8.79 ± 4.39**
		By family request		Mean ± SD	**120.28 ± 10.02**	**77.80 ± 6.11**	**29.08 ± 3.14**	8.66 ± 1.27	**67.21 ± 29.19**	7.13 ± 3.42	**18.90 ± 8.17**	**12.30 ± 5.22**		**13.50 ± 6.56**	**6.75 ± 3.22**	**8.68 ± 4.48**
		Teacher guidance		Mean ± SD	**124.81 ± 6.91**	**80.63 ± 4.45**	**30.18 ± 2.48**	9.18 ± 0.87	**50.27 ± 28.12**	5.09 ± 3.36	**13.45 ± 7.31**	**9.36 ± 5.35**		**10.09 ± 5.75**	**5.81 ± 2.96**	**6.27 ± 4.45**
		Other		Mean ± SD	**122.51 ± 8.16**	**79.53 ± 4.88**	**29.53 ± 3.41**	8.67 ± 1.30	**77.08 ± 26.55**	7.62 ± 3.15	**21.44 ± 8.05**	**13.84 ± 4.50**		**15.37 ± 5.76**	**7.91 ± 3.12**	**10.62 ± 3.82**
Kruskal Wallis test					**12.899**	**13.178**	**14.858**	2.434	**13.167**	7.448	**12.624**	**13.153**		**9.462**	**10.419**	**15.428**
*p* value					***p* = 0.005**	***p* = 0.004**	***p* = 0.002**	*p* = 0.487	***p* = 0.004**	*p* = 0.059	***p* = 0.006**	***p* = 0.004**		***p* = 0.024**	***p* = 0.015**	***p* = 0.001**
Effect size (d)					**0.2503**	**0.2173**	**0.1485**	–	**0.3015**	–	**0.2198**	**0.2403**		**0.1955**	**0.1941**	**0.2544**
**Liking the nursing profession and clinical practice**		Yes	Mean ± SD		**124.54 ± 8.81**	**80.51 ± 5.22**	**30.42 ± 3.12**	8.78 ± 1.23	67.18 ± 29.85	6.90 ± 3.34	18.86 ± 8.44	11.90 ± 5.38	13.43 ± 6.56		6.88 ± 3.34	9.02 ± 4.48
		No	Mean ± SD		**121.77 ± 9.03**	**78.19 ± 5.79**	**30.11 ± 3.38**	8.72 ± 1.27	77.33 ± 26.64	7.83 ± 3.50	21.44 ± 8.44	14.00 ± 4.68	15.66 ± 5.73		7.80 ± 2.99	10.50 ± 3.79
		Undecided	Mean ± SD		**121.15 ± 9.21**	**78.41 ± 5.58**	**29.19 ± 3.28**	8.80 ± 1.29	68.11 ± 27.97	7.03 ± 3.14	19.24 ± 8.02	12.26 ± 5.06	13.72 ± 6.02		6.89 ± 3.13	8.96 ± 4.21
Kruskal Wallis test					**10.550**	**14.952**	**8.312**	0.142	3.688	2.726	3.594	4.672	3.341		2.482	3.270
*p* value					***p* = 0.005**	***p* = 0.001**	***p* = 0.016**	*p* = 0.932	*p* = 0.158	*p* = 0.256	*p* = 0.166	*p* = 0.097	*p* = 0.188		*p* = 0.289	*p* = 0.195
Effect size (d)					**0.1766**	**0.2004**	**0.1011**	–	–	–	–	–	–		–	–
**Cigarette use**		Yes	Mean ± SD		123.85 ± 9.57	79.84 ± 5.75	30.50 ± 3.27	8.71 ± 1.22	**60.57 ± 31.60**	6.30 ± 3.62	17.30 ± 9.21	11.28 ± 5.92	**11.78 ± 6.93**		**5.97 ± 3.22**	**7.80 ± 4.76**
		No	Mean ± SD		123.22 ± 8.92	79.68 ± 5.39	29.95 ± 3.21	8.80 ± 1.25	**70.66 ± 28.10**	7.23 ± 3.20	19.76 ± 8.04	12.48 ± 5.04	**14.27 ± 6.10**		**7.25 ± 3.22**	**9.53 ± 4.18**
Mann–Whitney U test					U = 8597.0	U = 8695.0	U = 8367.0	U = 8738.5	**U = 7552.5**	U = 7808.0	U = 7851.0	U = 8210.5	**U = 7230.5**		**U = 7147.0**	**U = 7242.5**
Z statistic					−0.758	−0.623	−1.086	−0.590	**−2.226**	−1.876	−1.808	−1.303	**−2.638**		**−2.810**	**−2.670**
*p* value					*p* = 0.449	*p* = 0.533	*p* = 0.278	*p* = 0.555	***p* = 0.026**	*p* = 0.061	*p* = 0.071	*p* = 0.192	***p* = 0.007**		***p* = 0.005**	***p* = 0.008**
Effect size (d)					**–**	–	–	–	**0.3590**	**–**	**–**	–	**0.4081**		**0.3975**	**0.4138**

^*^p < 0.005, SD: Standard deviation, Mann–Whitney U test; Independent sample t test (non-parametric test), Kruskall Wallis test; One-way ANOVA (non-parametric test), NSPBS: Nursing Students Professional Behaviors Scale, PSSNS: Perceived Stress Scale for Nursing Student

When the total and all sub-dimensions of the NSPBS and descriptive variables were evaluated, it was observed that the mean scores of the total and "Activity Practices" sub-dimension scores of the students whose reason for choosing the nursing profession was teacher guidance were significantly higher than the others. The mean scores of those who liked the nursing profession and clinical practices were significantly higher than the others (*p* < 0.05). It was determined that the mean scores of the students in the 4th grade in the "Health Care Practices" sub-dimension were significantly higher than the others. The mean scores of those who chose the nursing profession because of teacher guidance were significantly higher than the others. The mean scores of those who liked the nursing profession and clinical practices were significantly higher than the others (*p* < 0.05) (Table 4). The difference between the mean scores of those who chose the nursing profession with teacher guidance and those who chose the nursing profession voluntarily was relatively small. In this case, while it was expected that students who chose nursing voluntarily would positively affect professional behaviors, it was not predictable that the professional behaviors of students who chose nursing with teacher guidance would be positively affected and high. In this context, students’interest in and commitment to nursing increased as they started clinical practice, which may have been reflected in professional professionalism. The fact that 4th-grade nursing students’healthcare practices were higher than those of the lower grades may be due to the high number and duration of clinical practices. In addition, the high professional behaviors of the students who love nursing show that their commitment to the profession and the behavior may be related to their interest and love for the profession.

The fact that the perceived stress in nursing students was higher in women may be thought to be because women experience their emotional anxiety more intensely and express it more and that men’s perceptions of stress are less than women’s. The fact that the perceived stress level of the students who chose nursing for other reasons was higher than the others may be interpreted as reluctance to the profession and concerns about making mistakes in professional practices. In addition, the high perceived stress levels of nursing students who do not smoke may be related to their anxiety about healthcare beliefs and practices and their search for positive coping with perceived stress.

Simple linear regression analysis was used to determine the predictive factors for nursing students’professional behavior and sub-dimensions. At this stage, considering the correlation relationships found to be significant, professional behavior could be a predictor of perceived stress total scores, and simple linear regression analysis was performed in this context. The alternative hypotheses of the regression analysis were formed as follows:

- H_1a_: As the perceived stress of nursing students increases, their professional behaviors decrease.
- H_**1b**:_ As the perceived stress of nursing students increases, healthcare practices decrease.
- **H**_**1c**_: As the perceived stress of nursing students increases, activity practices decrease.
- **H**_**1d**_: As the perceived stress of nursing students increases, the level of reporting practices decreases.

The findings of the simple linear regression analysis conducted in this context are summarized in Table 5.

**Table 5:** Predictive results of regression analysis for nursing students’professional behavior-sub dimensions

Variables		β	t	p	F	Model (p)	_R_2
Dependent Variables	Independent Variables						
**Professional Behavior**	**Constant**	126.87	100.82	< 0.001	**9.215**	**0.003**	**0.027**
	**PSSNS**	**−0.051**	**−3.036**	**0.003**			
**Health Care Practices**	**Constant**	81.102	105.87	< 0.001	3.866	0.050	0.012
	**PSSNS**	−0.020	−1.966	0.050			
**Activity Applications**	**Constant**	**31.981**	**72.593**	** < 0.001**	**22.147**	** < 0.001**	**0.063**
	**PSSNS**	**−0.028**	**−4.706**	** < 0.001**			
**Reporting**	**Constant**	8.988	51.210	< 0.001	1.611	0.205	0.005
	**PSSNS**	−0.003	−1.269	0.205			

*p* < 0.05: Statistically significant, ß: Standardized coefficients, F: ANOVA, R^2^: Adjusted R square, Statistical analysis: Linear Regression, *NSPBS* Nursing Students Professional Behaviors

According to the findings in Table 5, the regression model established to test whether professional behavior is affected by perceived anxiety level was found statistically significant (F = 9.215, *p* = 0.003; F = 22.147, p < 0.001). According to the same findings, hypotheses H_1b_ and H_1d_ were rejected, and it can be said that healthcare practices and reporting of practices are not related (β = −0.020, t = −1.966,* p* = 0.050; β = −0.003, t = −1.269, *p* = 0.205). According to this regression model, hypotheses H_1a_ and H_1c_ are accepted. As the perceived stress of nursing students increases, the level of professional behavior and activity practices increases negatively, decreasing r (β = −0.051, t = −3.036, *p* = 0.003; β = −0.028, t = −4.706, p < 0.001). In addition, nursing students’perceived stress explained their professional behaviors by approximately 0.3% (R^2^ = 0.027) and their activity practices by approximately 0.6% (R^2^ = 0.063).

Reasons such as acute situations encountered in clinical practice, clinical environment, and frequent clinical rotations may increase the perceived stress levels of nursing students. This situation may negatively affect nursing students’students’professional behaviors in clinical practice and patient care. For students who feel comfortable in clinical practice, this may be a supportive factor in the professional approach to patient care. In this context, it reveals the necessity of supporting nursing students in reducing anxiety and stress by the instructors who guide them in clinical practice and clinical guide nurses in practice.

## Discussion

Nursing students may face many daily academic, social, individual, or familial stressors, which may affect professional care in clinical practice. This study discussed the effect of stress perceived by nursing students in clinical practice on professional behavior in line with the literature.

### Discussion of results related to perceived stress

As a result of the study, the mean total PSSNS scale score of nursing students was found to be 68.53 ± 29.12. Accordingly, it can be said that the perceived stress in students is at a moderate level (Table 2). Similar results were obtained in studies on nursing students [1, 42]. Nursing students may feel conflict due to inconsistency between the disease and the intended learning outcomes during clinical practice [20]. This situation may cause stress and affect students’students’clinical practice performance. In addition, students’exposure to different stressors may negatively affect their professional identity development, mental health, problem-solving skills, critical thinking, and critical decision-making.

When evaluated in terms of gender, it was found that female students had higher mean scores in the total score and all sub-dimensions of PSSNS (Table 4). Similar results were obtained in studies on nursing students [10, 43]. However, the difference between genders may be explained by female students being more emotionally sensitive and expressing their emotions more than males. Contrary to our findings, studies also report no significant difference between gender and stress level or higher stress levels in male students [29, 44]. This difference may be due to socio-cultural differences between the sample groups.

A significant difference was found between the stress perceived by nursing students and the reasons for choosing the profession. This difference was higher in students who chose the profession for"other"reasons (Table 4). In contrast to our study, in Koras Sözen’s study, although the perceived stress level was higher in students who chose the profession at the request of their family, no statistically significant difference was found between the reasons for choosing a profession [24]. In our study, concerns about employment may be among the"other"reasons related to students ’students’ choice of profession. In this context, the differences between the studies may be due to differences in the student population.

It was found that there was a significant difference between smoking and perceived stress in nursing students, and the stress level was low in students who smoked (Table 4). In contrast to our study, Yörük reported that the perceived stress level was significantly higher in university students who smoked [47]. Studies have reported that nursing students use cigarettes and electronic cigarettes to reduce stress [3, 30]. Beliefs and experiences about smoking may have played a role in the development of a positive or negative attitude towards smoking in the following processes. In addition, the high level of stress perceived by non-smokers may be related to the inability to use healthy coping methods.

### Discussions about professional behavior

The mean NSPBS scale total score of nursing students was 123.36 ± 9.05. Accordingly, it can be said that the students exhibit a high level of professional behavior (Table 2). The findings of similar studies on the subject are in parallel with our study [6, 14, 27]. In our study, the NSPBS health care practice sub-dimension mean score of senior nursing students was higher than other grades. When the studies are examined, most studies show that professionalism increases as the grade level increases [5, 22]. This difference can be explained by the fact that senior students have a high perception of care, their awareness of clinical practices, professional knowledge, and skills have increased, and they take an active role in patient care because they are in the internship education process. However, it was also thought that as the education process progressed, theoretical knowledge increased, new skills were acquired, and this process was reflected in patient care.

A statistically significant difference was found between the professional behaviors of nursing students and the reasons for choosing the profession. This difference was higher in students who chose the profession with "their own will or teacher guidance" and was relatively small (Table 4). It has been reported that nursing students’choice of profession relates to perspective, beliefs, and values towards the profession and volunteering [37]. In this context, studies have reported that the professional care behaviors and professional commitment of those who choose a profession by their own choice are higher than those who choose a profession with family guidance or environmental influence [12, 26, 33].

A statistically significant difference was found between liking the nursing profession and clinical practice and professional care behaviors (Table 4). It has been reported that students who like to work in practice areas have higher levels of individualizing and supporting patient care [11]. This may be associated with the student’s love for the profession and willingness to sacrifice themselves in the clinical practice environment. In addition, it was thought that students who liked the nursing profession adopted it more and internalized nursing care, which was reflected in clinical practice as professional care behavior.

### Correlation and predictors between perceived stress and professional behavior

A low level and negative correlation was found between the students’total NSPBS scores, total PSSNS, and all sub-dimension scores (Table 3). In other words, as the stress level perceived by students increases, their professional behaviors are negatively affected. Similar results were obtained with our study results [4, 45]. In our study, while a high relationship was expected between students’perceived stress levels and their professional behaviors, a lower relationship was found. This may be related to students’students’professional beliefs and perceptions, coping levels with stressors, and problem-solving skills. In addition, socio-cultural differences between the students and differences related to temperament or perspective on the profession were considered adequate in our findings.

The regression analysis determining the effect of perceived stress levels on the professional behavior of nursing students was found to be statistically significant (Table 5). Perceived stress in the clinical practice process was determined as a reason for decreased professional behavior. Although clinical practice is an environment where knowledge and skills are developed for nursing students, it is also an important source of stress [23]. In this context, some problems students encounter during clinical practice may be a source of stress for them. For example, factors such as interpersonal problems, concerns about the practice, anxiety about making mistakes, changing rotations during the practice process, adaptation problems to each new clinic, off-duty demands of clinical nurses, etc., may cause stress in students. This situation may negatively affect adaptation to the clinic, nursing practices, and professional approach. In this context, responsible instructors must create alternatives to help students cope with the stressors they may encounter during clinical practice.

## Conclusion

This study revealed that perceived stress was effective on professional care behavior in nursing students. Stress level was found to be significantly different in terms of gender, choice of profession and smoking. At the same time, professional behaviors differed according to the student’s grade level, the reason for choosing a profession, and liking the profession. It is also critical to determine which other parameters affect perceived stress levels in addition to professional behavior. In this context, the findings can be expanded by conducting more detailed interviews and qualitative studies for future research. In order to identify the factors that cause stress in students and to reduce stress sources, it should be ensured that the necessary protective and preventive approaches are planned by focusing on individual needs.

By examining the effects of perceived stress in the clinic on the professional development of nursing students, this study provides important findings for increasing stress management and professional behaviors in nursing education. In this context, in line with the results of the research, recommendations for nursing, especially psychiatric nursing, are as follows:

- It is important to provide nursing students with stress management training to cope with the stressors they may encounter during clinical practice. These trainings can positively affect students’students’professional behaviors by enabling them to cope with stress more effectively.
- In the clinical setting, mentoring and guidance systems provided by experienced nurses or faculty members should be strengthened to help students cope with their challenges. This support will not only help students cope with stress but also enable them to improve their professional behavior.
- An environment where students can receive emotional support in clinical practice should be provided. This will reduce their stress levels and help them to make clinical decisions more healthily. Furthermore, emotional support can contribute to developing basic professional skills such as empathy and professionalism.
- Comprehensive psycho-educational programs for nursing students addressing stress’s effects on professional behaviors should be organized. These programs help students minimize the negative effects of stress and healthily build their professional identities.
- The intensity of clinical practice can increase students’students’stress levels. Therefore, it is necessary to plan clinical experiences in a balanced way and provide more support for students to feel psychologically ready. In addition, students should be provided with stress-coping strategies to strengthen their educational process before starting clinical practice.

In conclusion, it was observed that the stress experienced by nursing students in clinical practice significantly affected their professional behaviors. Improvements in educational processes and strengthening support mechanisms to minimize the negative effects of stress will contribute to the professional development of nursing students.

## Data Availability

All data supporting the findings of this study are included in the article. For further questions, please contact the corresponding author.
